# Rochester Pathological Society (Continued)

**Published:** 1886-08

**Authors:** Edward B. Angell


### *ROCHESTER PATHOLOGICAL SOCIETY.*

(CONTINUED.)

REPORTED BY EDWARD B. ANGELL, M. D.

DR. S. S. LATTIMORE, Professor of Chemistry in the University of Rochester, read a paper on Urea, its physiological chemistry, with a method of quantitative analysis. The following is a very brief synopsis of the paper:

It is a fact equally interesting to the chemist and physiologist, that urea was the first of the so-called organic substances which was produced artificially or outside of the animal organism. It was believed that an impassable line separated organic and inorganic bodies; that while the latter might be produced by artificial synthesis, the former could originate only in the presence—if not by the intervention—of vital forces. This conclusion was reasonable, because in strict accordance with all the facts observed. But in 1828, Wöhler, in Berlin, succeeded in producing, artificially, the first compound, in all its properties, identical with a body previously known only as a product of animal organism. This was urea, a compound of the following composition, expressed centesimally:

| | PER CENT. |
|---|---|
| Carbon, - - - - | 20.00 |
| Hydrogen, - - - - | 6.67 |
| Nitrogen, - - - - | 46.67 |
| Oxygen, - - - - | 26.66 |
| | 100. |

As is well known, stimulated by the success of Wöhler, other chemists have succeeded in producing a vast number of organic compounds, many of which are of much greater practical value than urea.

It is for the physiologist, rather than the chemist, that urea possesses the highest interest. In his view, the animal organism is an engine which receives various organic compounds, and oxidizes them with oxygen derived from the air. It is in this change, from low to high oxidation, that energy is liberated While animals seem capable of accepting an almost indefinite variety of compounds in their dietary, the chief forms under which, after serving their purpose, they are eliminated, are but three—water, carbon di-oxide and urea. These are the final products of vital activity. Carbon di-oxide is a gas, completely oxidized, and eliminated chiefly by the lungs. Water, the most highly oxidized body known, is eliminated chiefly in the liquid form by the lungs, the skin, and the kidneys. Urea is an incompletely oxidized body, eliminated, almost exclusively, by the kidneys. Though a solid, crystallizable body, it is exceedingly soluble. In an adult, the ingesta and egesta must constitute an exact equation. The difference in form constitutes an index to the changes through which they have passed in the hidden processes of the organism. The quantity of each, eliminated, becomes significant. Of the three final products just mentioned, the urea is the only one which can be readily collected and determined with accuracy. The value of such determination appears, when we remember that probably the entire nitrogen—that element which so peculiarly belongs to the animal kingdom—which is received in food, is eliminated in the form of urea, which contains nearly half its weight of that important element, and constitutes 80 per cent. of the total solids of the urine. The rate of tissue waste, whether normal or pathological, is registered in the quantity of urea produced. The elimination of this body must keep close step with its generation, since its accumulation in the blood, to an extent only slightly beyond the normal, produces incipient uremia. All pathological conditions involving increase of tissue waste, are indicated by an increase in the quantity of urea produced; hence the diagnostic value afforded by this knowledge. The value of a qualitative determination of albumen or sugar in the renal secretion is obvious—these substances in any quantity indicating disease. It is otherwise with urea. A qualitative analysis is of no significance, as it is always present in both normal and abnormal conditions; hence, we are driven to the difficult task of a quantitative analysis, if we are to make use of the valuable indications of the incipiency or progress of disease, or of the efficacy of remedies in restoring the equilibrium of opposing forces. The desirability of securing this information has been of late years widely felt, and has led to the invention of a multitude of instruments and methods of greater or less complexity. The best of these methods yet devised depends on the principle, that upon completely oxidizing urea by suitable re-agents, which affect oxidation indirectly, it is wholly converted into water, carbon di-oxide and nitrogen. Fortunately, no other constituents of urine, at least in any considerable quantity, are affected by such re-agents, so that no preliminary separation of the urea is required. The water immediately assumes the liquid form; the carbon di-oxide may be completely absorbed by an alkaline solution, leaving the whole of the nitrogen in the pure state as a gas. It only remains to ascertain the *weight* of nitrogen evolved from a known *weight* of urine, in order to ascertain the quantity of urea which it holds in solution, it being remembered that the nitrogen constitutes 26 per cent. of the weight of urea.

The writer urged that, at least from a theoretical point of view, even slight fluctuations in the quantity of urea should, if possible, be sought for diagnostic purposes, as preceding such signs as increase of temperature or pulse rate, and he condemned such methods as can only claim to be approximately quantitative. An apparatus capable of yielding exact results, is usually complex, and requires both skill and time to use it, and the latter condition generally places it out of the reach of the busy practitioner, who can rarely claim an uninterrupted hour.

Dr. Lattimore then presented to the society a form of *Ureometer*, which was the result of some years of practical experience on the the part of himself, and also of the students in his chemical laboratory. No claim of originality is made for the apparatus, except in the simple combination of well-known devices, by which both rapidity of execution and accuracy of results are secured. The objective point was to produce an apparatus of simple and inexpensive form, which the busiest practitioner could find time to use, so that a complete and exact quantitative analysis can be executed in the space of a very few minutes; and even an assistant, ignorant of the scientific principles involved, could soon be trained to use it successfully. The process is briefly this: A measured quantity of urine is decomposed by sodium hypobromide, which is formed at the moment of use, the resulting nitrogen gas being collected in a graduated burette. This is connected at the lower end with a sliding glass reservoir filled with water, by which the pressure can be instantly and precisely equalized. The volume of nitrogen is then taken. A glance at a table of figures gives the correction for the existing temperature and barometric pressure. Then a glance at a second table furnishes the percentage *by weight* of the urea contained in the sample of urine analyzed. If the quantity of urine secreted in twenty-four hours is ascertained, the total daily quantity of urea excreted can at once be easily calculated—the whole process occupying but a few minutes of time. We regret that we cannot present a cut of the apparatus, which, we are well aware, is necessary to a clear understanding of the apparatus which we have rather outlined than described. The accuracy of the instrument was shown by performing, in the presence of the society, an analysis on a solution containing a known percentage of artificial urea.

Dr. Damis, of Rochester, reported a case of progressive muscular atrophy, and subsequently presented the patient, James L. N., aged 48; occupation, contractor, formerly a nurseryman. Family history good. Temperate in all things. Has led an active out-door life until three years ago. Had considered himself an unusually healthy man until ten years ago, with this exception, viz.: About fifteen years ago, he was thrown violently from a carriage to the ground, striking upon the back of his left shoulder, head and neck. Was picked up in an insensible condition, and remained unconscious for a little more than an hour Both arms were partially paralyzed, though this was only a transient symptom.

He continued well for four years, with the exception of slight pain and stiffness in the neck and shoulders; and now came on an attack of what the physicians, whom he consulted, called, "Dumb ague." He had chills, slight fever, pains in different parts of the body, marked mental and physical depression, but no actual loss of muscular power anywhere. Appetite was indifferent, but the bowels were regular. No vertigo or other head symptoms. Under treatment, these symptoms disappeared, with the exception that he remained mentally depressed without any apparent cause, and had but little appetite. His mental depression was his most annoying symptom, yet he continued his business until the winter of 1880 and 1881, at which time he was traveling through the West. After a long ride, when the thermometer was 23° below zero, he had a severe chill; several hours elapsed before re-action came on. A day or two after this, he noticed that his right hand was uncomfortably cold, while the rest of his body was usually warm. Another peculiar symptom became at this time manifest: a burning sensation in the right cheek and eye, which, to use his own words, "seemed as if there was inflammation in them." That sensation has continued uninterruptedly from that time till now. About this same time, he noticed also that he could not hold a pen readily in his cold hand. The fingers seemed stiff, so that he would have to warm his hand before he could write. After a few weeks, he saw that he was actually losing the strength and grip of that hand; and this feebleness gradually increased, till in about six months there was complete inability to hold anything in the hand for the purpose; he would drop his knife in eating, etc.

Within the following six months, this progressive muscular wasting invaded the arm, so that he could not raise his hand to his head to take off his hat, or to put his hand in his pocket In a little more than one year from the time of his exposure to cold, the muscles of the hand, fore-arm, arm and shoulder were wasted to mere strings; the largest muscle mass remaining around the elbow.

The wasting began in the adductors of the thumb, and, almost simultaneously, in the muscles between the metacarpal bone of the thumb and that of the index finger. During all this time, there was absolute freedom from pain, while the tactile sense was perfect. Nor was there absolute paralysis of motion, while, at the present time, the attenuated muscles respond to the will as well as their crippled condition will admit.

During all this time, the left hand and arm were unaffected, *i. e.*, until the muscles of the right shoulder were well wasted. Then the flexors of the left thumb began to waste, as they had done in the right a year before; and this wasting and loss of contractility took the same course identically as did their counterparts of the other member; so that it has now been about eighteen months since the patient has been able to use either hand or arm to any extent. If there is any difference between the two, it is in favor of the last affected, because with the left hand he can convey food to his mouth. This he does by resting the elbow upon the edge of the table, and using this as a fulcrum, he can bend the elbow a little; and by bending the body forward still more, he can approximate the mouth and fingers. When the elbows are not so rested, his arms fall as dead weights to his sides. The muscles of the upper part of the trunk are quite progressed in atrophy, and I think the muscles of the neck are becoming involved. During the last three months, the patient has experienced more or less cramping and pains in the feet and legs. But about the only difficulty in locomotion, is that of a sense of weakness in the legs, although the muscles here do not show any marked signs of wasting.

At times, he has, as he expresses it, twitching of the muscles of the trunk, especially in the abdominal region. The last is becoming more and more pronounced. His organic functions seem to be going on fairly well. Appetite is poor, but food does not distress him. Bowels regular; power over sphincters unimpaired.

Within the last ten days, the patient has had some new symptoms, viz.: a drumming in the ears, with tenderness behind and around the right ear; also has a twitching of eyelids; eyesight has failed somewhat during past year, and especially so during the last month. I do not know that any opthalmoscopic examination has been made. Query—Are these prodromes of progressive bulbar paralysis?

So much, then, as to the history proper of this, to me, interesting case. Before I close, however, I desire to call your attention briefly to another morbid phenomenon manifest in the person of this much-afflicted man.

In the course of, and evidently attached to, the right sterno-mastoid muscle, is a growth, irregular in contour, having two sharply-defined prominences, the whole mass being quite movable and yet quite firmly imbedded in a nidus of muscle. This growth is fully equal in area to a fair-sized hen's egg, but is much longer.

He does not know just how long it has been there, but thinks not less than two years. I have examined it carefully, and I am satisfied that it is nothing more or less than bone. It has no connection whatever with any portion of the osseous system.

It is painless, giving no trouble, except as it limits somewhat rotation and flexion of the head.

If it is bone, it is the only case I have seen of a bony formation in muscle, but it may not be new to some of you. Exostoses, of course, are common enough, but independent osseous deposits like this, I think, are extremely uncommon, inasmuch as by a pretty thorough search through some seven standard works on surgery, I was not able, except in one, to find even a mention of anything like it.

As illustrating the extent to which such bony formations may occur, I find a record of a case which was admitted into St. George's Hospital in 1843, in which the patient, a young man of twenty-two, was literally incased in an armor of bone. The record says: "The greater part of the latissimus dorsi on either side was ossified; large masses of bone filled up to the hollows on either side of the vertebral spines from the sacrum to the occiput, soldering all the bones together into an inflexible column.

"The ribs were immovable by ossific deposits in the intercostal muscles, so that respiration was performed entirely by the diaphragm. The trapezins and the deep muscles at the sides of the neck contained large deposits of bone; the scapulas were immovably fixed to the ribs by ossification of the serratus magnus and rhomboid muscles. The pectoral muscles were ossified in their entirety."

This man finally died, because of extensive bony deposits in the muscles of deglutition and respiration. "The causes of osseous growth in muscles," says Mr. Clarke, "are not easily explained." He quotes Aberneth as mentioning the case of "a lad in whom either an exostosis, or bony growth in muscle, invariably followed a blow on the part." You will remember the fall upon the nape of the neck which this man had fifteen years ago. Remember it, please, in connection with his atrophy, and also in connection with this growth, and decide for yourselves how much credit, as an etiological factor, is to be accorded to it in this case.

A careful examination also discloses considerable spinal curvature in the cervical region, which is significant of the probable extent of the injury caused by the fall. Whether or not this was the origin of the difficulty, the peculiar grouping and progress of the symptoms point inevitably to alteration in the anterior-motor cells of the cord.

Dr. L. A. Weigel, of Rochester, presented a paper on the "Restoration of Crippled Joints," and gave the history of fibrous ankylosis, involving several joints. Complete restoration of their function was effected by *brisement force*.

